# COVID-19: The Need for Rational Use of Face Masks in Nigeria

**DOI:** 10.4269/ajtmh.20-0433

**Published:** 2020-05-15

**Authors:** Dimie Ogoina

**Affiliations:** Department of Internal Medicine, Infectious Disease Unit, Niger Delta University/Niger Delta University Teaching Hospital, Bayelsa, Nigeria

## Abstract

Because of the pandemic of COVID-19, the federal government of Nigeria has instituted a mandatory policy requiring everyone going out in public to wear face masks. Unfortunately, the Nigeria media is awash with images of misuse and abuse of face masks by the public, government officials, and healthcare workers. Medical masks are used widely in community settings amid reported scarcity within healthcare facilities. It is observed that some people wear face masks on their chin and neck, and mask wearers give no attention to covering their mouth and nose, especially when talking. Used face masks are kept with personal belongings or disposed indiscriminately in public spaces, leading to self and environmental contamination. Inappropriate use and disposal of face masks in Nigeria could promote the spread of the novel coronavirus in the country and negate the country’s efforts to contain the COVID-19 pandemic. In the implementation of the universal masking policy in Nigeria, federal and state governments ought to consider local applicability, feasibility, and sustainability, as well as identify and mitigate all potential risks and unintended consequences. Also critical is the need for intensive public sensitization and education on appropriate use and disposal of face masks in the country.

The major strategic goals of COVID-19 control efforts are to slow or stop transmission and spread of SARS-COV-2 and to mitigate the impact of the virus on the health system, social activities, and economies of countries and communities.^1^ The scientific information on SARS-COV-2 and COVID-19 is rapidly evolving, and many countries are adopting preventive measures based on emerging evidence and local applicability. One such strategy, considering evidence of presymptomatic and asymptomatic transmissions of SARS-COV-2, is the use of face masks by apparently healthy persons to slow community transmission of the virus.^2–4^

The use of public face masks for the prevention of COVID-19 is controversial. The WHO has indicated that it cannot recommend for or against public use of face masks, as there is yet no clear evidence that this practice is effective in the prevention of COVID-19.^5^ The WHO has also emphasized that “the use of a mask alone is insufficient to provide an adequate level of protection, and other measures should also be adopted.” However, the WHO acknowledged potential advantages of the use of masks by healthy people in the community setting to reduce potential exposure from infected persons during the presymptomatic period of infection. It advised a risk-based approach for implementing policies on public wearing of face masks.^5^

As part of a comprehensive response to the COVID-19 epidemic, the president of the Federal Republic of Nigeria recently announced mandatory wearing of face masks by anyone going out in public.^6^ A similar policy is being implemented by almost all state governments of the country. The Nigerian Centre of Disease Control (NCDC) indicated that the major rationale for public wearing of face masks is to prevent those who are infected but asymptomatic from spreading the virus.^7^ The NCDC has emphasized that wearing of face masks may only be effective in preventing the transmission of SARS-COV-2 if they are worn and disposed appropriately, and if mask wearing is combined with other preventive measures such as hand hygiene and social distancing. An advisory on making and proper usage of cloth masks has also been issued by the NCDC; it was recommended that cloth masks be used by the general public, with medical masks reserved for healthcare workers.^8^

Unfortunately, new face mask policies are leading to widespread misuse and abuse of face masks in Nigeria.^9–12^ The Nigeria media is awash with images of members of the general public, including healthcare workers and government officials, wearing face masks on their jaws and neck, without covering their mouth or nose, or covering only their mouth while the nose is left opened. Many people who use face masks are commonly observed to pull down their mask to their jaw to talk and then pull it back over their mouth and nose after talking. A variety of cloth masks of doubtful efficacy are hawked on the streets and tried by different wearers before deciding on purchase.

People are also observed to repeatedly touch the front of their face masks in a bid to adjust the mask, to remove it, or during reflex touching of the face. Some wear one mask for prolonged periods, without replacement when it is wet or soiled. Furthermore, face masks used for the prevention of COVID-19 by the general public are being disposed inappropriately.^11^ The rising spate of misuse and abuse of face masks is a source of worry for the Nigerian COVID-19 Presidential Task Force, which observed “unhygienic and ill-advised use and sharing of masks, especially multiple fittings before buying from vendors.”^13^

It is noteworthy that medical masks meant for healthcare workers, such as surgical masks and respirators, are being routinely worn by the general public and government officials, when there are complaints that these masks are not available in sufficient quantities in Nigerian hospitals.^14^ Furthermore, N95 respirators with exhalation valves have become the preferred face masks by many, including top government officials, possibly because they are more comfortable. Respirators with exhalation valves are, however, not effective in COVID-19 source control, as they do not prevent the release of exhaled respiratory particles.

Although some state governments in Nigeria are now producing large quantities of cloth masks for use by the general population,^10^ one is concerned if sufficient quantities of these homemade masks can be provided sustainably for a population of more than 200 million. There are also challenges related to enforcement of mandatory usage, access to water and soap to properly wash and reuse homemade masks, and a false sense of security that may lead people to abandon other preventive measures because of the usage of face masks. Compliance with and enforcement of social distancing measures have been identified as challenges in Nigeria’s response to COVID-19, and there are reports of people openly flouting lockdown orders and other preventive measures.^15^ Many Nigerians still do not have access to water and basic sanitation, and indices of hygiene are poor in the country.^16^

Adopting a public face mask strategy may require extra funding to be sustainable, and this strategy could divert scarce resources from other COVID-19 preventive measures. Another concern is that face masks might become a new medium for propagation of the novel coronavirus in Nigeria in view of high risks of self and environmental contamination when masks are used and disposed inappropriately.

Public mask wearing is most effective at stopping the spread of the novel coronavirus when compliance is high and when masks are used appropriately, especially in combination with other preventive measures such as hand hygiene.^5,17^ In many of its public health advisories, including the advisory on use of face masks by the healthy community,^5^ the WHO cautioned on universal applicability of public health measures without consideration of local context. In the absence of an effective and intensive communication strategy on why, when, and how to use face masks, the strategy of mandatory public face mask use in Nigeria carries risks and uncertainties. The federal and state governments ought to consider the local peculiarities, resource requirements, feasibility, sustainability, and potential risks and benefits before and during the implementation of public face mask policies. Failure to address these may negate the potential benefits of public face mask policies and inadvertently enhance the spread of SARS-COV-2 in Nigeria.
